# Antibodies to Citrullinated Protein Antigens, Rheumatoid Factor Isotypes and the Shared Epitope and the Near-Term Development of Clinically-Apparent Rheumatoid Arthritis

**DOI:** 10.3389/fimmu.2022.916277

**Published:** 2022-06-22

**Authors:** Dylan T. Bergstedt, Wyatt J. Tarter, Ryan A. Peterson, Marie L. Feser, Mark C. Parish, Christopher C. Striebich, M. Kristen Demoruelle, LauraKay Moss, Elizabeth A. Bemis, Jill M. Norris, V. Michael Holers, Jess D. Edison, Geoffrey M. Thiele, Ted R. Mikuls, Kevin D. Deane

**Affiliations:** ^1^ Department of Medicine, St. Joseph’s Hospital, SCL Health, Denver, CO, United States; ^2^ Division of Rheumatology, University of Colorado Denver Anschutz Medical Campus, Aurora, CO, United States; ^3^ Department of Biostatistics and Informatics, Colorado School of Public Health, University of Colorado-Denver Anschutz Medical Campus, Aurora, CO, United States; ^4^ Department of Epidemiology, Colorado School of Public Health, University of Colorado Denver Anschutz Medical Campus, Aurora, CO, United States; ^5^ Department of Medicine, Walter Reed National Military Medical Center, Bethesda, MD, United States; ^6^ University of Nebraska Medical Center and VA Nebraska-Western Iowa Health Care System, Omaha, NE, United States

**Keywords:** rheumatoid arthritis (RA), pre-rheumatoid arthritis (pre-RA), antibodies to citrullinated protein antigens (ACPA), rheumatoid factor (RF), prediction of future rheumatoid arthritis, shared epitope (SE)

## Abstract

**Background/Purpose:**

In rheumatoid arthritis (RA) autoantibodies including antibodies to citrullinated protein antigens (ACPA) and rheumatoid factor (RF) can be predictive of incident clinical RA. However, there is limited understanding of how antibody changes over time impact prediction of the likelihood and timing of future clinical RA.

**Materials and Methods:**

We evaluated relationships between ACPA, the shared epitope (SE), RF isotypes and incident RA in a prospective cohort of 90 ACPA(+) individuals without baseline arthritis identified through health-fair testing (i.e. Healthfair). We also evaluated ACPA and RF isotypes and time-to-diagnosis of RA in a retrospective cohort of 215 individuals with RA from the Department of Defense Serum Repository (DoDSR).

**Results:**

Twenty-six of 90 (29%) of ACPA(+) Healthfair participants developed incident RA. Baseline or incident dual RF-IgA and RF-IgM positivity was associated with increased risk for incident RA (HR 3.09; 95% CI 1.15 to 8.29) although RFs were negative in ~50% of individuals with incident RA. SE was associated with increased risk of RA (HR 2.87, 95% CI 1.22-6.76). In the DoDSR cohort, triple positivity for ACPA, RF-IgA and RF-IgM was present a median of 1-2 years prior to RA diagnosis, with some sex-specific differences.

**Conclusion:**

These findings can be used to counsel individuals at-risk for future RA and to design clinical trials for RA prevention. The findings also suggest that RF could be a surrogate outcome as a success of an immunologic intervention in RA prevention. Additional studies are needed to understand the biologic of different patterns of autoantibody elevations in RA evolution.

## Introduction

A number of studies demonstrate that there is a period of seropositive rheumatoid arthritis (RA) development that can be termed ‘Pre-RA’ during which there are elevations of circulating autoantibodies including antibodies to citrullinated protein antigens (ACPA) and rheumatoid factor (RF) in absence of and prior to the appearance of clinically-apparent inflammatory arthritis (IA) as well as a clinical diagnosis of RA (clinical RA) that may further classifiable by established criteria (1–3). Importantly, these autoantibodies may play a pathogenic role in the development of RA (4, 5); furthermore, the diagnostic accuracy of these autoantibodies for the future onset of clinical IA/RA has underpinned the development of several clinical prevention trials (1, 6–10).

A key aspect of these trials is to use as a component of the inclusion criteria a biomarker profile that is highly predictive for future RA onset (i.e. likelihood of RA) as well as incident RA within a defined time interval to optimize clinical trial design and duration by having highly accurate estimates of expected incidence rates.

Notably, some published data suggest that combinations of ACPA and RF are highly predictive of future RA within a relatively short time period (11–15). In addition, several studies have reported that the presence of the shared epitope (SE) in the setting of ACPA positivity is associated with higher risk of progression to future IA/RA (16, 17). However, many prospective studies evaluating the prediction of future RA have only utilized autoantibody positivity at a single time point or not found conclusive improvements in prediction based on changing autoantibody levels over time (14, 18–20). As such, there is a limited understanding of how longitudinal changes of autoantibody positivity for ACPA and RF may further inform the likelihood and timing of incident clinical IA/RA, as well as potentially provide insights into how various ‘endotypes’ of RA may develop (e.g. ACPA and RF positive RA, versus ACPA positive alone). To address this gap, herein we have utilized two separate cohorts to evaluate the role of autoantibody positivity over time, as well as the presence of the SE, to define the likelihood and timing of incident clinical IA/RA.

## Materials and Methods

### Study Populations

Two separate cohorts were used in these analyses. The first cohort was created in Colorado from individuals identified with ACPA positivity through health-fair based testing and is termed the ‘Healthfair’ cohort. As described previously, at a series of Colorado-based health-fairs, individuals who did not have a prior diagnosis of RA were offered the opportunity for blood testing for ACPA (17, 21). Individuals who were positive for the ACPA test anti-cyclic citrullinated peptide (anti-CCP3, Inova Diagnostics Inc., San Diego, CA) were invited to an additional follow-up research visit. If at that visit they were confirmed to be ACPA(+) on repeat testing and did not have prior or current clinically-apparent IA/RA, they were enrolled into a longitudinal follow-up study where questionnaires were administered, serial joint examinations performed (66/68 count by a rheumatologist or trained personnel) and serial autoantibody biomarker testing was performed. Incident clinical IA/RA was identified at scheduled research visits or at *ad hoc* visits if there were changing symptoms, and individuals with IA were classified as having RA by the 2010 American College of Rheumatology/European League Against Rheumatism (ACR/EULAR) criteria (2). Notably, none of the Healthfair cohort was treated with disease modifying anti-rheumatic therapy prior to the onset of incident RA.

The second cohort is a retrospective case-control cohort created from the Department of Defense Serum Repository (DoDSR) and is termed the ‘DoDSR cohort’. The DoDSR is part of a program to monitor the health of US military personnel (22–24) and the creation of the cohort of RA cases and controls that is used herein has been previously described (25–27). In brief, 215 individuals who had a diagnosis of clinical RA were identified based on documentation in the medical record and at least one rheumatologist encounter, and confirmation of diagnosis by medical chart review by a rheumatologist or trained rheumatology nurse from Walter Reed National Military Medical Center (WRNMMC), with 212 (~99%) of cases meeting 1987 RA classification criteria. Material for genetic studies was not available from the DoDSR. Notably, we have previously used this DoDSR cohort to evaluate the relationship between various biomarkers including ACPA. A single isotype of RF (IgM) and calprotectin and the timing of a future diagnosis of RA (27). However, we are including this cohort in these new analyses to validate the findings in the Healthfair cohort, and furthermore we will present new analytic approaches and biomarker findings (e.g. combinations of RF-IgA and RF-IgM isotypes) not previously reported in this cohort.

### Autoantibody Testing

Serum samples from the Healthfair and DoDSR cohorts were tested using enzyme linked immunoabsorbent assays (ELISA) for anti-cyclic citrullinated peptide-3 (anti-CCP3 IgG, Inova Diagnostics Inc., San Diego, CA), and RF-IgA and RF-IgM isotypes (QUANTA Lite platform, Inova Diagnostics Inc., San Diego, CA). Notably, we did not evaluate RF-IgG given it is not widely available for routine clinical testing. All autoantibody testing was performed at the University of Colorado in the Exsera Biolabs, with the technician blinded to the case-control status of samples. Anti-CCP3 positivity was evaluated based on the manufacturer established cut-off of ≥20 units. Following a guideline from the 1987 classification criteria for RA (3), RF-IgA and RF-IgM positivity was determined based on levels present in <5% of two control groups. Specifically, for the Healthfair cohort, we determined the RF cut-offs in a group of 491 randomly selected blood donors from Colorado. For the DoDSR cohort, we used a group of 156 controls selected from the DoDSR who did not have a diagnosis of RA based on chart review; furthermore, these controls were matched to the RA cases on age, sex, race and region of enlistment in the military (26).

### Shared Epitope Testing

Genetic material was only available from the Healthfair cohort and it was typed for the presence of HLA alleles containing the shared epitope (SE) using methods previously described (28). Participants were considered SE positive (dichotomous variable yes/no) if one or more allele included the following subtypes: DRB1*0401, *0404, *0405, *0408, *0409, *0410, *0413; *0101, *0102 and *1001.

### Statistical Analyses

#### Healthfair Cohort

We evaluated baseline characteristics between participants who did or did not develop incident IA/RA using Fishers exact test or two sample t-tests as appropriate, and computed descriptive transition rates between different RF positivity statuses for all samples. In addition, we created graphical representations of progression to RA based on baseline factors (e.g. autoantibodies) using Kaplan-Meier curves. For our main analysis, we present time-to-RA from study entry as an outcome in a series of Cox regression models with a time-varying covariate denoting baseline or incident positivity for autoantibodies, with adjustment for SE status and anti-CCP3 levels <=60/>60 units. Differences in IA-free probabilities are tested *via* log-rank tests with type I error rate of 0.05. Finally, we plotted predicted survival curves under several realistic hypothetical trajectories from baseline to repeat testing at 1 year and accounting for changes in various anti-CCP3 and RF isotype states (and stratified by the presence/absence of the SE) using the technique of Smith and colleagues (29).

#### DoDSR Cohort

Given this cohort was retrospectively created and all cases developed RA we did not utilize it to replicate exactly the analyses in the prospective Healthfair cohort; instead, we focused on analyses that evaluated the relationship between combinations of autoantibodies and the timing of a future diagnosis of RA. We produced summary statistics for variables of interest, and sex-based differences at each sample collection time were conducted using Fisher’s Exact tests. For each sample, the time-to-RA was calculated and is presented stratified by positivity status in boxplots. For inference between these strata, time-to-RA was treated as a time-to-event variable and modeled *via* a Cox regression with positivity status as a time-varying covariate (a Markov renewal model), thus the hazard of developing RA after each measurement is assumed to be independent of previous encounters. Additionally, these models are stratified by (e.g. a different baseline hazard estimated for) the number of pre-RA diagnosis samples each person had in the data set to account for the fact that certain patients did not have all measurements. The output of this method is hazard ratios; the factor increase in the hazard of developing RA for each 1-unit increase (or positivity) in each covariate, holding other covariates constant. Finally, to assess pairwise group differences in the time-to-RA among those who had: 1) no positivity, 2) anti-CCP3 positivity, 3) any RF positivity, or 4) anti-CCP3 and dual RF-IgA and RF-IgM positivity, we used a series of pairwise Wald tests. These tests are adjusted for differences in age and gender, and the p-values are adjusted for multiple comparisons using the false discovery rate method of Benjamini-Hochberg (30). Aside from these latter pairwise comparisons, nominal (unadjusted) p-values are presented in the results.

### Ethical Considerations

Study activities using the DoDSR data and samples were approved by institutional review boards at the University of Colorado and WRNMMC, and study activities using the Healthfair data and samples were approved by institutional review board at the University of Colorado.

## Results

### Healthfair Cohort

#### Descriptive Characteristics

The descriptive characteristics of the Healthfair cohort are reported in **Table 1**. Of the 90 subjects, 26 (29%) developed incident IA/RA after a mean of 731 days (~2 years) and over a mean of 1111 days (~3 years) of follow-up of the entire cohort. All 26 (100%) of those with incident IA met 2010 ACR/EULAR classification criteria for RA at the time of initial identification of their IA.

**Table 1:** Characteristics of the Healthfair cohort.

	No incident IA/RA (n=64)	Incident IA/RA (n=26)	P-value
Days to incident IA/RA or last follow-up visit, mean (SD)	1265 (887)	731 (836)	-
Age at baseline visit, mean (SD)	58 (12)	55 (12)	0.263
Age at diagnosis of IA/RA, mean (SD)	-	57 (11)	-
Number of total visits or number of visits prior to incident IA/RA, mean (SD)	5 (3)	3 (2)	<0.001
Female, n (%)	39 (61%)	20 (77%)	0.221
Non-Hispanic white, n (%)	54 (84%)	20 (77%)	0.600
At least 1 allele containing the shared epitope, n (%)	24 (38%)	18 (69%)	0.005
Ever smoker (Baseline visit), n (%)	24 (38%)	11 (42%)	0.812
Current smoker (Baseline visit), n (%)	3 (5%)	1 (4%)	0.114
Self-reported number of painful joints (Baseline visit), median (range)	0 (0-18)	1 (0-24)	0.142
Self-reported presence of >=1 painful joint (Baseline visit), n (%)	30 (47%)	18 (69%)	0.065
Anti-CCP3 positive at standard cut-off level (>=20 units) at baseline visit, n (%)	64 (100%	26 (100%)	1.000
Anti-CCP3 >2 times the upper limit of normal (>40 units) at baseline visit, n (%)	39 (60%)	22 (85%)	0.045
Anti-CCP3 >3 times the upper limit of normal (>60 units) at baseline visit, n (%)	24 (38%)	17 (65%)	0.020
Anti-CCP positive at last visit, or visit prior to incident IA/RA, n (%)	55 (86%)	26 (100%)	0.055
Anti-CCP3 >2 times the upper limit of normal at last visit or visit prior to incident IA/RA, n (%)	40 (63%)	20 (77%)	0.224
Anti-CCP3 >3 times the upper limit of normal at last visit or visit prior to incident IA/RA, n (%)	26 (41%)	16 (62%)	0.102
RF patterns at baseline visit, n (%) RF-IgA(-) RF-IgM(-) RF-IgA(-) RF-IgM(+) RF-IgA(+) RF-IgM(-) RF-IgA(+) RF-IgM(+)	49 (77%) 11 (17%) 2 (3%) 2 (3%)	17 (65%) 3 (12%) 0 (0%) 6 (23%)	0.301 0.749 1.000 0.007
RF patterns at last visit, or visit prior to incident IA/RA, n (%) RF-IgA(-) RF-IgM(-) RF-IgA(-) RF-IgM(+) RF-IgA(+) RF-IgM(-) RF-IgA(+) RF-IgM(+)	44 (69%) 10 (16%) 5 (8%) 5 (8%)	13 (50%) 6 (23%) 1 (4%) 6 (23%)	0.226 0.543 0.668 0.145
Autoantibody patterns at or after developing incident IA/RA, n (%) Anti-CCP3 positive standard cut-off (>=20 units) Anti-CCP3 >2 x upper limit of normal (>40 units) Anti-CCP3 >3x upper limit of normal (>60 units) RF-IgA(-) RF-IgM(-) RF-IgA(-) RF-IgM(+) RF-IgA(+) RF-IgM(-) RF-IgA(+) RF-IgM(+)	n/a	26/26 (100%) 23/26 (89%) 18/26 (69%) 14/26 (54%) 5/26 (19%) 1/26 (4%) 6/26 (23%)	n/a

IA, inflammatory arthritis; RA, rheumatoid arthritis; SD, standard deviation; anti-CCP, anti-cyclic citrullinated peptide; RF, rheumatoid factor; Ig, immunoglobulin; n/a, not applicable.Bold means statistically significant results (i.e. p < 0.05).

#### Baseline Factors and Incident IA/RA

In univariate analyses, compared to individuals who did not develop incident IA/RA, at their baseline visit the individuals who developed incident IA/RA had a higher prevalence of positivity for at least one allele containing the shared epitope, a higher prevalence of an anti-CCP3 level >2 and >3 times the upper limit of normal as well as a higher prevalence of positivity for both RF-IgA and RF-IgM (**Table 1**). There were no significant associations at the baseline visits between incident RA and the presence/absence of joint pain or smoking status (**Table 1**). In addition, at baseline the prevalence of RF-IgM positivity was significantly higher in current and ever smokers, although the prevalence of RF-IgA positivity was not (**Supplemental Table 1**).

**Figure 1:**
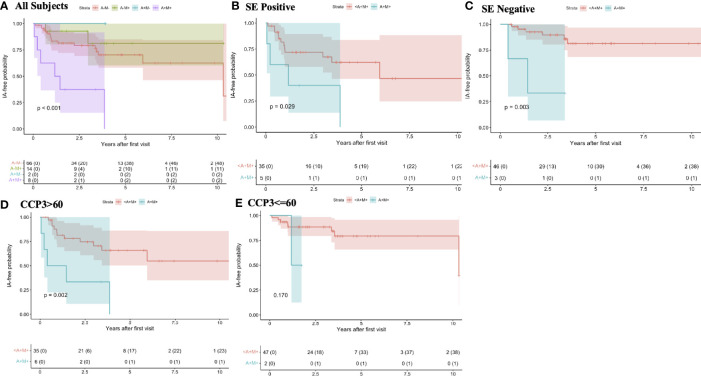
Rates of progression to inflammatory arthritis/rheumatoid arthritis by baseline rheumatoid factor isotype positivity and stratified by shared epitope positivity and baseline anti-CCP3 levels In this cohort, at baseline, all individuals are anti-CCP3 positive at the standard cut-off (>=20 units). In all subjects **(A)** the individuals who were additionally dual positive at baseline for RF-IgA and RF-IgM (purple line) had a significantly greater rate of progression to IA/RA than individuals who were positive for only one RF isotype (blue and green lines), or who were negative for both (red line). In individuals stratified by the presence **(B)** and absence **(C)** of at least one allele containing the shared epitope, baseline dual positivity for RF-IgA and RF-IgM was associated with increased rate of progression to IA/RA (**B**, green lines) compared to individuals who were positive for only one RF isotype or who were negative for both isotypes (**B**, red lines). The lowest incidence rate of IA/RA was in participants who were SE negative and who did not have dual positivity for RFIgA and RF-IgM (**C**, red line). In individuals who had a baseline anti-CCP3 level of >60 units (3 times the upper limit of normal), baseline dual positivity for RF-IgA and RF-IgM was associated with increased rate of progression to IA/RA **(D)**, green line. There was a similar trend in those with anti-CCP3 levels <=60, although this was not statistically significant **(E)**. The colored bands around each line represent 95% confidence intervals. A, rheumatoid factor IgA; M, rheumatoid factor IgM; <A+M+, positive for RFIgA or RFIgM, or neither but not both; SE, shared epitope; IA, inflammatory arthritis; RA, rheumatoid arthritis; RF, rheumatoid factor; CCP, anti-cyclic citrullinated peptide antibody; Ig, immunoglobulin.

In survival models and Kaplan-Meier curves there was a significantly higher incidence of IA/RA in individuals who at baseline were dual positive for RF-IgA and RF-IgM when compared to those who were positive for only one RF isotype, or no RF isotypes (**Figure 1A**). In addition, because the presence of an anti-CCP3 level of >60 units was associated with increased risk for RA in univariate analysis, and that high level is also given additional points towards RA classification in the 2010 ACR/EULAR criteria, and the presence of the SE was also associated with increased risk for incident IA/RA (**Table 1**), we further evaluated the relationship between RF positivity and incident IA stratified by baseline anti-CCP3 levels (<=60 or >60), and the presence/absence of the SE (**Figures 1B–E**). In these analyses, in both SE positive and negative individuals the incidence of IA was significantly higher in individuals who were dual RF-IgA and RF-IgM positive (**Figures 1B, C**), although the lowest incidence of IA/RA was in individuals who were SE negative and did not have at baseline dual positivity for RF-IgA and RF-IgM (**Figure 1C**). In addition, in participants with baseline anti-CCP3 levels >60, the incidence of IA/RA was significantly greater in those with dual positivity for RF-IgA and RF-IgM (**Figure 1D**). However, in participants with baseline anti-CCP3 levels of <=60, while the survival curves visually differed, there were no significant differences in IA/RA incidence between those who developed dual positivity for RF-IgA and RF-IgM (**Figure 1E**).

#### Longitudinal Biomarker Changes and Incident IA/RA

Descriptions of autoantibody positivity at the last follow-up visit or visit immediately prior to incident IA/RA are presented in **Table 1**, and in more detail in **Supplemental Table 2** and **Supplemental Figure 1**. Overall, most (>50%) of individuals and samples maintained their original pattern of autoantibody positivity over time. However, there were non-significant trends for the individuals who did not develop IA/RA to have lower prevalence of autoantibody positivity than those who developed incident IA/RA. In particular, 9/64 (14%) individuals who did not develop IA/RA lost positivity for anti-CCP3 compared to 0/26 (0%) in those who developed incident IA/RA (p>0.05).

To address the effect of changing autoantibody positivity over time on incident IA/RA, we used a Cox regression model and a time-varying covariate to evaluate the role of baseline and incident RF positivity and risk for incident IA/RA, and adjusting for the presence of the shared epitope and anti-CCP3 level positive at >60. In these analyses (the results of which are presented in detail in **Supplemental Table 3**) baseline or incident dual RF-IgA and RF-IgM positivity was associated with a significantly higher risk for incident IA/RA (Hazard Ratio 3.09, 95% Confidence Interval 1.15 to 8.29, p=0.025). The presence of the SE was also significantly associated with increased risk for RA (HR 2.87, 95% CI 1.22 to 6.76, p=0.016); however, positivity for only one RF isotype (RF-IgA or RF-IgM) not associated with a significantly increased risk for incident IA/RA (RF-IgA positive only: HR 1.20, 95% CI 0.16 to 9.32; RF-IgM positive only: HR 1.33, 95% CI 0.47 to 3.78, p=0.5990). In contrast to the univariate analyses, in these multivariate analyses, positivity for anti-CCP3 >60 was not significantly associated with incident RA (HR 1.45, 95% CI 0.62 to 3.39, p=0.390).

**Figure 2:**
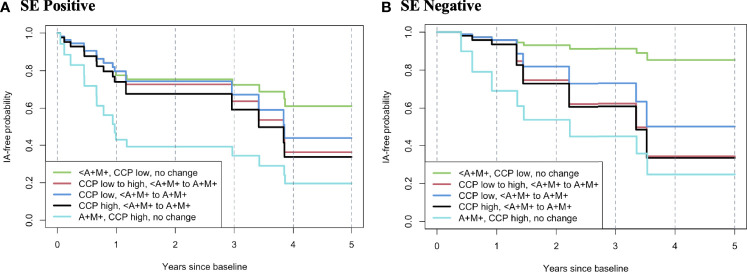
Hypothetical model of rates of progression to inflammatory arthritis/rheumatoid arthritis based on change of autoantibody profile from baseline to 365 days. In this model, all individuals are anti-CCP3 positive at baseline. The rates of progression to IA/RA are modelled using data from the Healthfair cohort and based on a change from a baseline state of autoantibody positivity to a state at 365 day s as this can approximate a clinical care pathway where an individual who has autoantibody positivity without IA/RA is re-evaluated for changes in autoantibody positivity at 1 year. The figures also present models stratified by positivity/negative for the shared epitope. Overall, the highest rate of progression to IA/RA was in individuals who were SE positive and had high anti-CCP3 (>60 units) and dual positivity for RF-IgA and RF-IgM at baseline that persisted at 365 days (**A**, light blue line), with the lowest rate of incident IA/RA in SE(-) individuals with baseline and follow-up low anti-CCP3 (<=60 units) and who were positive for one or less RF isotype (**B**, green line). A, rheumatoid factor IgA; M, rheumatoid factor IgM; <A+M+, positive for RFIgA or RFIgM, or neither but not both; SE, shared epitope; IA, inflammatory arthritis; RA, rheumatoid arthritis; RF, rheumatoid factor; CCP, anti-cyclic citrullinated peptide antibody; Ig, immunoglobulin.

We also created hypothetical models to visualize the relationships between various ‘states’ of autoantibody positivity at baseline as well as at a repeat visit at 1 year, as this could approximate a clinical situation. In these analyses, individuals who were positive for the SE and persistently positive at baseline and 1 year for anti-CCP3 >60 units, and dual RF-IgA and RF-IgM had the highest rate of incident clinical IA/RA (**Figure 2A**). Individuals that transitioned at 1 year from antibody negative to positive (either double RF-IgA and RF-IgM, CCP high, or both), had higher rates of incident clinical IA/RA than the negative at baseline group, while also having lower incidence than hypothetical individuals that were antibody positive from baseline (**Figures 2A**, **B**). In contrast, individuals who had the lower incidence of RA were negative for the SE, persistently had an anti-CCP3 level of <=60 and were persistently negative for RF-IgA and RF-IgM (**Figure 2B**).

**Figure 3:**
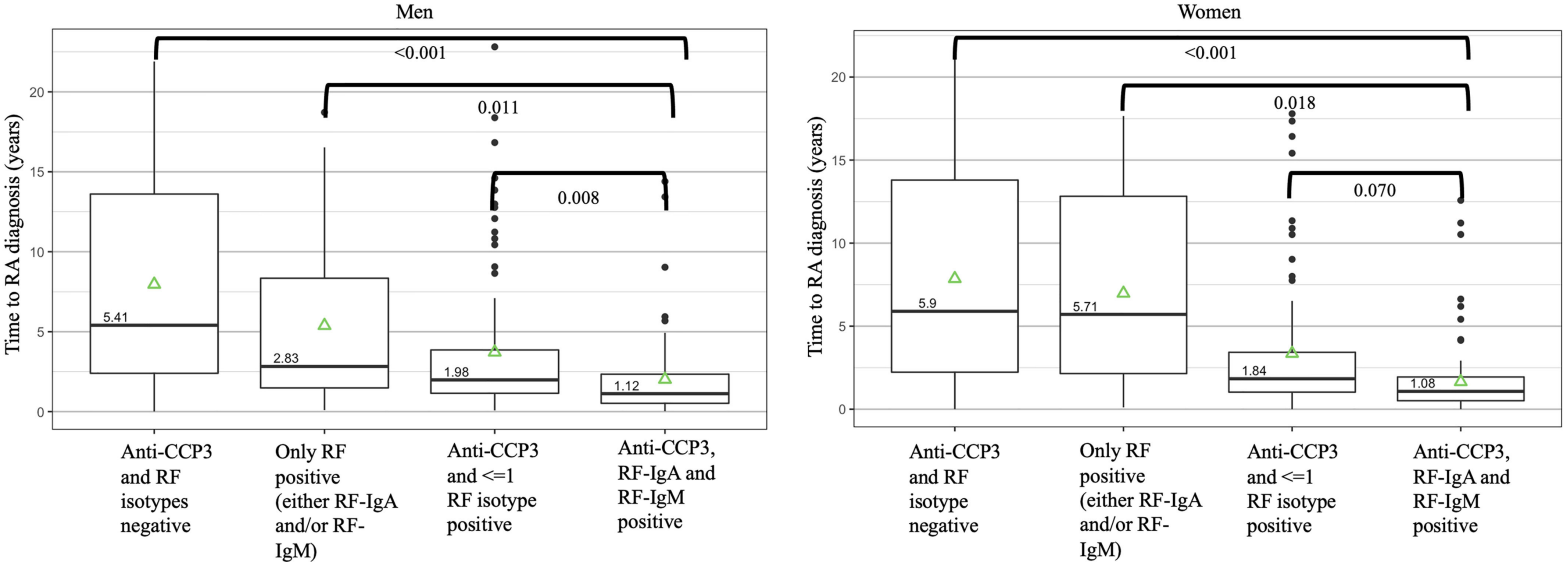
Autoantibody positive states and median time to a future diagnosis of rheumatoid arthritis in the Department of Defense Serum Repository cohort. The times to diagnosis are stratified by men (n=113) and women (N=103) as women had a higher overall prevalence of rheumatoid factor (RF) positivity than men. Overall, positivity for anti-CCP3, RF-IgA and RF-IgM in a sample was seen closest to diagnosis. Of note, while not in the figure, in men, anti-CCP3 positivity at >60 units (with or without positivity for ≤1 RF isotype) was present a median of 1.93 years prior to diagnosis; in women, anti-CCP3 positivity at >60 units (with or without positivity for ≤1 RF isotype) was present a median of 1.64 years prior to diagnosis. P-values represent comparisons between autoantibody positive states using pairwise contrasts and age-adjusted Cox regression model as well as adjusting using the false-discovery method of Benjamini-Hochberg. The green triangles represent the mean time of autoantibody positivity prior to RA diagnosis. DoDSR, Department of Defense Serum Repository; RA, rheumatoid arthritis; RF, rheumatoid factor; anti-CCP, anti-cyclic citrullinated peptide antibody; Ig, immunoglobulin.

### DoDSR Cohort

We also evaluated the relationship between anti-CCP3, RF-IgA and RF-IgM positivity and the timing of incident IA in the DoDSR cohort that is described in **Supplemental Table 4**. Notably, this cohort differed from the Healthfair in that pre-RA samples were selected retrospectively from individuals with a known ‘future’ diagnosis of RA and therefore we could not evaluated likelihood of future RA; furthermore, in the DoDSR cohort the earliest or ‘baseline’ visit, an individual did not have to be positive for anti-CCP3. In addition, compared to the Healthfair cohort, the participants in the DoDSR cohort had a higher percentage of males, the age of diagnosis of RA is younger, and there was less clinical data available including smoking status, and no genetic tests were available. Moreover, we identified in the DoDSR cohrt that women had a higher prevalence than men of RF-IgA and RF-IgM positivity at the earliest available time point pre-RA diagnosis as well as a higher prevalence of RF-IgA and RF-IgM positivity post-RA diagnosis (**Supplemental Table 4**), although there were no sex-specific differences in autoantibody positivity in the Healthfair cohort (**Supplemental Table 5**).

In these analyses (**Figure 3**), in women, samples that were negative for anti-CCP3 and both RF isotypes were a median of 5.90 years from a diagnosis of RA compared to samples that were ‘triple’ positive for anti-CCP3, RF-IgA and RF-IgM that were a median of 1.08 years prior to a diagnosis of RA. In men, samples that were negative for anti-CCP3 and RF were a median of 5.41 years from a diagnosis of RA compared to samples that were triple positive for anti-CCP3, RF-IgA and RF-IgM that were a median of 1.12 years prior to a diagnosis of RA.

## Discussion

In the prospectively evaluated Healthfair cohort of anti-CCP3 positive subjects without IA at baseline, we have identified that baseline or incident dual positivity for RF-IgA and RF-IgM is indicative of a subset of individuals who have a greater likelihood of developing near-term incident IA/RA. Importantly, this was true for ‘all comers’ who were anti-CCP3 positive at baseline at standard cut-off levels, as well as in individuals stratified by at baseline by the presence of either high-positive anti-CCP3 levels or the SE, although the loss of significance of an associations of high positive anti-CCP3 levels in multivariate analyses suggest that the dual positivity for RFs and SE are stronger predictors of incident IA/RA. Furthermore, in the DoDSR cohort ‘triple’ positivity of anti-CCP3, RF-IgA and RF-IgM was present closer to diagnosis. In aggregate, these findings support that a combination of positivity of anti-CCP3 and these two RF isotypes, including persistent ‘dual’ positivity for these RFs over time, is strongly associated with the future onset of clinical IA/RA, as well as imminent RA, with additional influence from the SE.

If an ACPA positive individual is identified who has these factors (e.g. dual RF isotype positivity, SE positivity, potentially high-positive ACPA), it may aid in counseling them as to their overall risk and potential timing of development of future IA/RA as well as referral to clinical rheumatologic care (15). In particular, the hypothetical model presented in **Figure 2** suggests that repeat evaluation for evolving autoantibody positivity at 1 year can be informative, and this may be a ‘real life’ clinical scenario and follow-up period. Furthermore, these findings may be applied going forward in clinical trial development for RA prevention to identify individuals who are at particularly high-risk for imminent onset of clinical IA/RA – and indeed several existing clinical prevention trials have as inclusion criteria either high-positive ACPA levels, or positivity for ACPA plus combinations of RF isotypes (7–9). Importantly, many prospective studies of pre-RA have utilized individuals who have initially presented to health-care with arthralgia and were subsequently found to have autoantibody positivity (14, 16); while the Healthfair cohort studied herein still had a substantial portion of individuals with some joint symptoms at baseline and therefore may be somewhat comparable to individuals identified through clinics, ~30% of ACPA(+) individuals who later developed RA did not report joint pain at baseline. As such, these findings suggest that approaches such as health-fair ACPA testing can identify individuals at higher risk for development of future RA, and these approaches may be incorporated into future clinical studies.

In addition, most of the current prevention trials in RA are using as primary endpoints clinical IA and classifiable RA. Those are reasonable outcomes given the appearance of clinical IA is currently a key clinical decision point in RA diagnosis and management. However, it may be that incident RF positivity could also be an important surrogate endpoint in preventive interventions in individuals who are ACPA positive. Specifically, while we do not yet know the complete pathophysiologic processes that may drive RF generation in pre-RA, ACPA and dual RF-IgA and RF-IgM positivity is likely indicative of an expansion of autoimmune processes towards a state where initiation of synovitis may be more likely and more imminent (4, 31). As such, an intervention that decreases prevalent or incident dual RF positivity in an ACPA positive individual may potentially decrease an overall risk for future RA. Supporting this notion, in the prospective Healthfair cohort the findings herein suggest that maintenance of RF negativity or the loss of RF positivity is associated with a ‘state’ that is at lower risk for progression to IA/RA – at least within the duration of the study. Moreover, these findings are similar to what has been described in a longitudinal study of a cohort of indigenous North American People where loss of ACPA and/or RF positivity occurred in individuals who did not develop incident IA/RA (18). Therefore, the ‘disappearance’ of RA-related autoantibody positivity may be truly associated with decreased risk for progression to clinical RA for some individuals.

A caveat, however, is that while autoantibodies are informative in identifying risk for future RA, autoantibody testing alone provides a limited understanding of the underlying pathophysiologic processes in RA development. In particular, ~77% of those who developed RA within the Healthfair cohort did not have dual RF-IgA and RF-IgM positivity, and an additional subset with incident RA were negative for both RF’s and/or had anti-CCP3 levels <=60. Furthermore, while SE was associated with incident RA, ACPA, RFs and incident RA still developed in SE negative individuals in the Healthfair cohort, and ~8% of those who did not develop incident RA were ACPA and dual RF-IgA and RF-IgM positive. Moreover, we have previously published that in the DoDSR cohort described herein a percentage (~20%) of individuals who developed clinical RA were positive for ACPAs and/or RF’s at some point in pre-RA yet lost positivity for at least one of those autoantibodies post-RA diagnosis (26). In aggregate, these points support that there are various ‘endotypes’ of RA risk and development that may be defined by autoantibodies and certain genetic factors (e.g. SE); however, these features are not comprehensive, and furthermore the loss of detectable autoantibodies may not be indicative of a reduced risk for future RA in all individuals. More broadly, these points highlight that additional studies are needed in order to understand the drivers of pathogenic autoimmune processes, autoantibody-related and otherwise (e.g. T cell autoreactivity), that are related to various aspects of RA development including early symptoms and transitions to clinical RA (4, 5, 32–34). These other factors may include environmental factors, mucosal and/or microbial influences (e.g. viral or bacterial) that importantly may also be targets for preventive interventions (33, 35, 36). Notably, in the Healthfair subjects smoking was associated with RF-IgM positivity but not RF-IgA, although smoking was not associated with incident RA; given prior studies associating smoking with RA-related autoantibodies as well as potentially incident RA (37), this will need further exploration.

Notably, the ACPA assay utilized herein was the anti-CCP3 assay and therefore it is not clear that findings herein are applicable to all ACPA assays which may have differing predictive values for future IA/RA (38, 39) In addition, there are multiple other factors including other autoantibody systems [e.g. antibodies to carbamylated antigens and/or other modified proteins (40)], inflammatory markers [e.g. C-reactive protein, serum calprotectin (27)], cytokines, chemokines and cellular assays (13, 34, 41) as well as clinical features such as joint symptoms (42) that may be incorporated into the prediction of the likelihood and timing of future IA/RA, and these will need further investigation.

A final item of interest was within the DoDSR cohort, women had a higher rate of positivity for RFs than men, although this was not the case in the Healthfair cohort. The reasons for this are not clear, and published studies of rates of RF positivity in patients with clinical RA are conflicting and often not reported in a sex-stratified manner (43). However, a consideration is that the mean age of diagnosis of RA in the DoDSR cohort was younger than most published cohorts, and indeed was ~20 years younger than the mean age at incident RA in the Healthfair cohort. With that, it may be that there is an age-related sex effect on RF development; this needs further exploration to understand the biology of RF development as well as potentially to develop more age and sex-specific prediction models for future RA.

In conclusion, in ACPA(+) individuals dual RF-IgA and RF-IgM positivity as well as the presence of the SE and can be an indicators of a higher likelihood and more imminent onset of clinical seropositive RA. Further studies are needed into the ‘endotypes’ of RA as well as the biologic relationships between ACPA, RFs, SE in the natural history of RA development.

## Data Availability Statement

The datasets presented in this article are not publicly available due to institutional review board requirements. Specific requests for data can be requested from corresponding author Kevin D. Deane. Requests to access the datasets should be directed to Kevin.deane@cuanschutz.edu.

## Ethics Statement

Study activities using the DoDSR data and samples were approved by institutional review boards at the University of Colorado and WRNMMC, and study activities using the Healthfair data and samples were approved by institutional review board at the University of Colorado. The patients/participants provided their written informed consent to participate in this study.

## Author Contributions

DB, RP, WT, and KD performed analyses and wrote the paper. MF, LM, and EB performed data and sample management. MP, MF, LM, and EB performed sample testing and results management. DB, MF, CS, MD, LM, EB, JN, VH, and KD recruited and evaluated subjects for the Healthfair cohort. MF, LM, EB, VH, JE, GT, TM, and KD constructed the DoDSR cohort and data. All authors contributed to the article and approved the submitted version.

## Funding

This project was supported by Congressionally Directed Medical Research Program (PR191079) (KD, VH, MF, TM, and GT) and an investigator-initiated grant from AbbVie (KD). The funder was not involved in the study design, collection, analysis, interpretation of data, the writing of this article or the decision to submit it for publication. This project was also supported by NIH/NIAMS P30 AR079369 (KD, VH, MF, and JN).

## Author Disclaimer

The identification of specific products or scientific instrumentation is considered an integral part of the scientific endeavor and does not constitute an endorsement or implied endorsement on the part of the author, the Department of Defense, or any component agency. The views expressed in this presentation are those of the authors and do not reflect the official policy of the Department of Army/Navy/Air Force, Department of Defense, or the United States Government.

## Conflict of Interest

KD has received free materials for autoantibody testing from Inova Diagnostics, Inc., and he has also served as an advisor for Inova Diagnostics, Inc.

The remaining authors declare that the research was conducted in the absence of any commercial or financial relationships that could be construed as a potential conflict of interest.

## Publisher’s Note

All claims expressed in this article are solely those of the authors and do not necessarily represent those of their affiliated organizations, or those of the publisher, the editors and the reviewers. Any product that may be evaluated in this article, or claim that may be made by its manufacturer, is not guaranteed or endorsed by the publisher.

## References

[1] Deane KD Holers VM. Rheumatoid Arthritis Pathogenesis, Prediction, and Prevention: An Emerging Paradigm Shift. Arth Rheumatol (2021) 73:181–93. doi: 10.1002/art.41417 PMC7772259 32602263

[2] Aletaha D Neogi T Silman AJ Funovits J Felson DT Bingham CO 3rd. 2010 Rheumatoid Arthritis Classification Criteria: An American College of Rheumatology/European League Against Rheumatism Collaborative Initiative. Arthritis Rheum (2010) 62(9):2569–81. doi: 10.1002/art.27584 20872595

[3] Arnett FC Edworthy SM Bloch DA McShane DJ Fries JF Cooper NS. The American Rheumatism Association 1987 Revised Criteria for the Classification of Rheumatoid Arthritis. Arthritis Rheumatol (1988) 31(3):315–24. doi: 10.1002/art.1780310302 3358796

[4] Sokolove J Johnson DS Lahey LJ Wagner CA Cheng D Thiele GM. Rheumatoid Factor as a Potentiator of Anti-Citrullinated Protein Antibody-Mediated Inflammation in Rheumatoid Arthritis. Arthritis Rheumatol (2014) 66(4):813–21. doi: 10.1002/art.38307 PMC3994896 24757134

[5] Kuhn KA Kulik L Tomooka B Braschler KJ Arend WP Robinson WH. Antibodies Against Citrullinated Proteins Enhance Tissue Injury in Experimental Autoimmune Arthritis. J Clin Invest (2006) 116(4):961–73. doi: 10.1172/JCI25422 PMC1421345 16585962

[6] Bos WH Dijkmans BA Boers M van de Stadt RJ van Schaardenburg D. Effect of Dexamethasone on Autoantibody Levels and Arthritis Development in Patients With Arthralgia: A Randomised Trial. Ann Rheum Dis (2010) 69(3):571–4. doi: 10.1136/ard.2008.105767 19363022

[7] Gerlag DM Safy M Maijer KI Tang MW Tas SW Starmans-Kool MJF. Effects of B-Cell Directed Therapy on the Preclinical Stage of Rheumatoid Arthritis: The PRAIRI Study. Ann Rheum Dis (2019) 78(2):179–85. doi: 10.1136/annrheumdis-2017-212763 PMC6352407 30504445

[8] Al-Laith M Jasenecova M Abraham S Bosworth A Bruce IN Buckley CD. Arthritis Prevention in the Pre-Clinical Phase of RA With Abatacept (the APIPPRA Study): A Multi-Centre, Randomised, Double-Blind, Parallel-Group, Placebo-Controlled Clinical Trial Protocol. Trials (2019) 20(1):429. doi: 10.1186/s13063-019-3403-7 31307535 PMC6633323

[9] van Boheemen L Turk S Beers-Tas MV Bos W Marsman D Griep EN. Atorvastatin Is Unlikely to Prevent Rheumatoid Arthritis in High Risk Individuals: Results From the Prematurely Stopped STAtins to Prevent Rheumatoid Arthritis (STAPRA) Trial. RMD Open (2021) 7(1):1–4. doi: 10.1136/rmdopen-2021-001591 PMC7942258 33685928

[10] Strategy for the Prevention of Onset of Clinically-Apparent Rheumatoid Arthritis (StopRA) (2018). Available at: https://clinicaltrials.gov/ct2/show/NCT02603146.

[11] Rantapaa-Dahlqvist S de Jong BA Berglin E Hallmans G Wadell G Stenlund H. Antibodies Against Cyclic Citrullinated Peptide and IgA Rheumatoid Factor Predict the Development of Rheumatoid Arthritis. Arthritis Rheumatol (2003) 48(10):2741–9. doi: 10.1002/art.11223 14558078

[12] Nielen MM van Schaardenburg D Reesink HW van de Stadt RJ van der Horst-Bruinsma IE de Koning MH. Specific Autoantibodies Precede the Symptoms of Rheumatoid Arthritis: A Study of Serial Measurements in Blood Donors. Arthritis Rheumatol (2004) 50(2):380–6. doi: 10.1002/art.20018 14872479

[13] Deane KD O'Donnell CI Hueber W Majka DS Lazar AA Derber LA. The Number of Elevated Cytokines and Chemokines in Preclinical Seropositive Rheumatoid Arthritis Predicts Time to Diagnosis in an Age-Dependent Manner. Arthritis Rheumatol (2010) 62(11):3161–72. doi: 10.1002/art.27638 PMC2980824 20597112

[14] van de Stadt LA Witte BI Bos WH van Schaardenburg D. A Prediction Rule for the Development of Arthritis in Seropositive Arthralgia Patients. Ann Rheum Dis (2013) 72(12):1920–6. doi: 10.1136/annrheumdis-2012-202127 23178208

[15] Garcia-Montoya L Nam JL Duquenne L Villota-Eraso C Di Matteo A Hartley C. Prioritising Referrals of Individuals at-Risk of RA: Guidance Based on Results of a 10-Year National Primary Care Observational Study. Arthritis Res Ther (2022) 24(1):26. doi: 10.1186/s13075-022-02717-w 35042555 PMC8767684

[16] Rakieh C Nam JL Hunt L Hensor EM Das S Bissell LA. Predicting the Development of Clinical Arthritis in Anti-CCP Positive Individuals With non-Specific Musculoskeletal Symptoms: A Prospective Observational Cohort Study. Ann Rheum Dis (2015) 74(9):1659–66. doi: 10.1136/annrheumdis-2014-205227 24728331

[17] Bemis EA Demoruelle MK Seifert JA Polinski KJ Weisman MH Buckner JH. Factors Associated With Progression to Inflammatory Arthritis in First-Degree Relatives of Individuals With RA Following Autoantibody Positive Screening in a non-Clinical Setting. Ann Rheum Dis (2021) 80(2):154–61. doi: 10.1136/annrheumdis-2020-217066 PMC7855648 32928740

[18] Tanner S Dufault B Smolik I Meng X Anaparti V Hitchon C. A Prospective Study of the Development of Inflammatory Arthritis in the Family Members of Indigenous North American People With Rheumatoid Arthritis. Arthritis Rheumatol (2019) 71(9):1494–503. doi: 10.1002/art.40880 30861615

[19] van Beers-Tas MH Stuiver MM de Koning MHMT van de Stadt LA Geskus RB van Schaardenburg D. Can an Increase in Autoantibody Levels Predict Arthritis in Arthralgia Patients? Rheumatology (Oxford) (2018) 57(5):932–4. doi: 10.1093/rheumatology/kex506 29401313

[20] Ten Brinck RM van Steenbergen HW van Delft MAM Verheul MK Toes REM Trouw LA. The Risk of Individual Autoantibodies, Autoantibody Combinations and Levels for Arthritis Development in Clinically Suspect Arthralgia. Rheumatology (Oxford) (2017) 56(12):2145–53. doi: 10.1093/rheumatology/kex340 PMC6703997 28968865

[21] Deane KD Striebich CC Goldstein BL Derber LA Parish MC Feser ML. Identification of Undiagnosed Inflammatory Arthritis in a Community Health Fair Screen. Arthritis Rheumatol (2009) 61(12):1642–9. doi: 10.1002/art.24834 PMC2913880 19950306

[22] Perdue CL Cost AA Rubertone MV Lindler LE Ludwig SL. Description and Utilization of the United States Department of Defense Serum Repository: A Review of Published Studies, 1985-2012. PLoS One (2015) 10(2):e0114857. doi: 10.1371/journal.pone.0114857 25723497 PMC4344338

[23] Perdue CL Eick-Cost AA Rubertone MV. A Brief Description of the Operation of the DoD Serum Repository. Mil Med (2015) 180(10 Suppl):10–2. doi: 10.7205/MILMED-D-14-00739 26444888

[24] Rubertone MV Brundage JF. The Defense Medical Surveillance System and the Department of Defense Serum Repository: Glimpses of the Future of Public Health Surveillance. Am J Public Health (2002) 92(12):1900–4. doi: 10.2105/AJPH.92.12.1900 PMC1447349 12453804

[25] Mikuls TR Edison J Meeshaw E Sayles H England BR Duryee MJ. Autoantibodies to Malondialdehyde-Acetaldehyde Are Detected Prior to Rheumatoid Arthritis Diagnosis and After Other Disease Specific Autoantibodies. Arthritis Rheumatol (2020) 72(12):2025–9. doi: 10.1002/art.41424 PMC7722146 32621635

[26] Kelmenson LB Wagner BD McNair BK Frazer-Abel A Demoruelle MK Bergstedt DT. Timing of Elevations of Autoantibody Isotypes Prior to Diagnosis of Rheumatoid Arthritis. Arthritis Rheumatol (2020) 72(2):251–61. doi: 10.1002/art.41091 PMC6994340 31464042

[27] Bettner LF Peterson RA Bergstedt DT Kelmenson LB Demoruelle MK Mikuls TR. Combinations of Anticyclic Citrullinated Protein Antibody, Rheumatoid Factor, and Serum Calprotectin Positivity Are Associated With the Diagnosis of Rheumatoid Arthritis Within 3 Years. ACR Open Rheumatol (2021) 3(10):684–9. doi: 10.1002/acr2.11309 PMC8516104 34288565

[28] Kolfenbach JR Deane KD Derber LA O'Donnell C Weisman MH Buckner JH. A Prospective Approach to Investigating the Natural History of Preclinical Rheumatoid Arthritis (RA) Using First-Degree Relatives of Probands With RA. Arthritis Rheumatol (2009) 61(12):1735–42. doi: 10.1002/art.24833 PMC2795101 19950324

[29] Smith A Goodrich N Beil C Liu Q Merion R Gillespie B. Graphical Representation of Survival Curves in the Presence of Time-Dependent Categorical Covariates With Application to Liver Transplantation. J Appl Stat (2019) 46:1702–13. doi: 10.1080/02664763.2018.1558187 PMC12034941 40297818

[30] Benjamini Y Hochberg Y. Controlling the False Discovery Rate: A Practical and Powerful Approach to Multiple Testing. J R Stat Soc (1995) 57:289–300. doi: 10.1111/j.2517-6161.1995.tb02031.x

[31] Lingampalli N Sokolove J Lahey LJ Edison JD Gilliland WR Holers VM. Combination of Anti-Citrullinated Protein Antibodies and Rheumatoid Factor Is Associated With Increased Systemic Inflammatory Mediators and More Rapid Progression From Preclinical to Clinical Rheumatoid Arthritis. Clin Immunol (2018) 195:119–26. doi: 10.1016/j.clim.2018.05.004 29842946

[32] Derksen VFAM Huizinga TWJ van der Woude D. The Role of Autoantibodies in the Pathophysiology of Rheumatoid Arthritis. Semin Immunopathol (2017) 39(4):437–46. doi: 10.1007/s00281-017-0627-z PMC5486798 28451788

[33] Rims C Uchtenhagen H Kaplan MJ Carmona-Rivera C Carlucci P Mikecz K. Citrullinated Aggrecan Epitopes as Targets of Autoreactive CD4+ T Cells in Patients With Rheumatoid Arthritis. Arthritis Rheumatol (2019) 71:518–28. doi: 10.1002/art.40768 PMC6438725 30390384

[34] Ponchel F Burska AN Hunt L Gul H Rabin T Parmar R. T-Cell Subset Abnormalities Predict Progression Along the Inflammatory Arthritis Disease Continuum: Implications for Management. Sci Rep (2020) 10(1):3669. doi: 10.1038/s41598-020-60314-w 32111870 PMC7048829

[35] Holers VM Demoruelle MK Kuhn KA Buckner JH Robinson WH Okamoto Y. Rheumatoid Arthritis and the Mucosal Origins Hypothesis: Protection Turns to Destruction. Nat Rev Rheumatol (2018) 14(9):542–57. doi: 10.1038/s41584-018-0070-0 PMC6704378 30111803

[36] Fechtner S Berens H Bemis E Johnson RL Guthridge CJ Carlson NE. Antibody Responses to Epstein-Barr Virus in the Preclinical Period of Rheumatoid Arthritis Suggest the Presence of Increased Viral Reactivation Cycles. Arthritis Rheumatol (2022) 74(4):597–603. doi: 10.1002/art.41994 34605217 PMC8957485

[37] Ishikawa Y Terao C. The Impact of Cigarette Smoking on Risk of Rheumatoid Arthritis: A Narrative Review. Cells (2020) 9(2). doi: 10.3390/cells9020475 PMC7072747 32092988

[38] Demoruelle MK Parish MC Derber LA Kolfenbach JR Hughes-Austin J Weisman MH. Performance of Anti-Cyclic Citrullinated Peptide Assays Differs in Subjects at Increased Risk of Rheumatoid Arthritis and Subjects With Established Disease. Arth Rheumatol (2013) 65:2243–52. doi: 10.1002/art.38017 PMC3776020 23686569

[39] Di Matteo A Mankia K Duquenne L Mahler M Corscadden D Mbara K. Third-Generation Anti-Cyclic Citrullinated Peptide Antibodies Improve Prediction of Clinical Arthritis in Individuals at Risk of Rheumatoid Arthritis. Arthritis Rheumatol (2020) 72(11):1820–8. doi: 10.1002/art.41402 32840033

[40] Verheul MK Böhringer S van Delft MAM Jones JD Rigby WFC Gan RW. Triple Positivity for Anti-Citrullinated Protein Autoantibodies, Rheumatoid Factor, and Anti-Carbamylated Protein Antibodies Conferring High Specificity for Rheumatoid Arthritis: Implications for Very Early Identification of At-Risk Individuals. Arthritis Rheumatol (2018) 70(11):1721–31. doi: 10.1002/art.40562 29781231

[41] Kokkonen H Soderstrom I Rocklov J Hallmans G Lejon K Rantapaa Dahlqvist S. Up-Regulation of Cytokines and Chemokines Predates the Onset of Rheumatoid Arthritis. Arthritis Rheumatol (2010) 62(2):383–91. doi: 10.1002/art.27186 20112361

[42] van Steenbergen HW Aletaha D Beaart-van de Voorde LJ Brouwer E Codreanu C Combe B. EULAR Definition of Arthralgia Suspicious for Progression to Rheumatoid Arthritis. Ann Rheum Dis (2017) 76(3):491–6. doi: 10.1136/annrheumdis-2016-209846 27991858

[43] Whiting PF Smidt N Sterne JA Harbord R Burton A Burke M. Systematic Review: Accuracy of Anti-Citrullinated Peptide Antibodies for Diagnosing Rheumatoid Arthritis. Ann Intern Med (2010) 152(7):456–64; W155-66. doi: 10.7326/0003-4819-152-7-201004060-00010 20368651

