# Supplementary material for: Antibodies to Citrullinated Protein Antigens, Rheumatoid Factor Isotypes and the Shared Epitope and the Near-Term Development of Clinically-Apparent Rheumatoid Arthritis
Source: Front Immunol. 2022 Jun 22;13:916277. doi: 10.3389/fimmu.2022.916277 (PMC9265214; doi:10.3389/fimmu.2022.916277)
Supplement: Supplementary file 1 [file DataSheet_1.docx]

Supplementary Material

| Supplemental Table 1. Relationships between smoking and rheumatoid factor positivity at baseline in the Healthfair cohort | | | |
| --- | --- | --- | --- |
|  | Ever smoke = yes  Total n=35 | Ever smoke = no  Total n=55 | p-value |
| **RF-IgM positive** | 13/35 (37%) | 9/55 (16%) | 0.043 |
| RF-IgA positive | 6/35 (17%) | 4/55 (7%) | 0.178 |
|  | Current smoker = yes  Total n=4 | Current smoker = no  Total n=80 | p-value |
| **RF-IgM positive** | 3/4 (75%) | 17/80 (21%) | 0.040 |
| RF-IgA positive | 2/4 (50%) | 8/80 (10%) | 0.068 |
| Abbreviations: RF=rheumatoid factor; Ig=immunoglobulin | | | |

| **Supplemental Table 2. Description of autoantibody profile changes over time in the Healthfair cohort** | | | | | | | |
| --- | --- | --- | --- | --- | --- | --- | --- |
|  |  | Intermediate visit(s) autoantibody profile (excludes final visits or visits immediately prior to incident IA/RA, but may include visits from individuals with or without incident IA/RA) | | | | Final visit if no incident IA/RA, or visit immediately prior to incident IA/RA | |
|  |  | Negative for RF-IgA and RF-IgM dual (+); anti-CCP3 (+) at <=60  N=95 samples | Negative for RF-IgA and RF-IgM dual (+); anti-CCP3 (+) at >60  N=110 samples | RF-IgA and RF-IgM dual (+); anti-CCP3(+) at <=60  N=3 samples | RF-IgA and RF-IgM dual(+), anti-CCP3(+) at >60  N=11 samples | Final visit in participants without incident IA/RA (n=64) | Visit immediately prior to IA/RA in those with incident IA (n=26) |
| Starting autoantibody profile | Negative for RF-IgA and RF-IgM dual (+); anti-CCP3 (+) at <=60  N=142 samples | 80/142 (56%) of samples | 16/142 (11%) of samples | 1/142 (<1%) of samples | 0/142 (0%) of samples | 36/142 (25%) of samples    36/64 (56%) of participants without IA/RA | 9/142 (6%) of samples    9/26 (35%) of participants) with IA/RA |
|  | Negative for RF-IgA and RF-IgM dual (+); anti-CCP3 (+) at >60  N=145 samples | 15/145 (10%) of samples | 93/145 (64%) of samples | 0/145 (0%) of sample | 3/145 (2%) of samples | 23/145 (16% of samples    23/64 (36%) of participants without IA/RA | 11/145 (8%) of samples    11/26 (42%) of participants with IA/RA |
|  | RF-IgA and RF-IgM dual (+); anti-CCP3(+) at <=60  N=5 samples | 0/5 (0%) of samples | 0/5 (0%) of samples | 2/5 (40%) of samples | 0/5 (0%) of samples | 2/5 (40%) of samples    2/64 (3%) of participants without IA/RA | 1/5 (20%) of samples    1/26 (4%) of participants with IA/RA |
|  | RF-IgA and RF-IgM dual(+), anti-CCP3(+) at >60  N=17 samples | 0/17 (0%) of samples | 1/17 (6%) of samples | 0/17 (0%) of samples | 8/17 (47%) of samples | 3/17 (18%) of samples    3/64 (5%) of participants without IA/RA | 5/17 (29%) of samples    5/26 (19%) of participants with IA/RA |
| Abbreviations: IA=inflammatory arthritis; RA=rheumatoid arthritis; RF=rheumatoid factor; Ig=immunoglobulin; anti-CCP3=anti-cyclic citrullinated peptide antibody | | | | | | | |

| Supplemental Table 3. Adjusted hazard ratios for incident IA/RA in the Healthfair cohort* using biomarkers in a Cox regression model and time varying covariates that account for changing autoantibody positivity over time. | | | |
| --- | --- | --- | --- |
|  | **HR** | **95% CI** | **p-value** |
| SE(+) | **2.87** | **1.22 to 6.76** | **0.016** |
| RF-IgA(-) RF-IgM(+) | 1.33 | 0.47 to 3.78 | 0.590 |
| RF-IgA(+) RF-IgM (-) | 1.20 | 0.16 to 9.32 | 0.860 |
| RF-IgA (+) and RF IgM(+) | **3.09** | **1.15 to 8.29** | **0.025** |
| Anti-CCP3 (+) at >60 units | 1.45 | 0.62 to 3.39 | 0.390 |
| *All subjects were positive at baseline for anti-CCP3 at the standard cut-off of >=20 units.  *Abbreviations: SE=shared epitope; HR=hazard ratio; CI=confidence interval; RF=rheumatoid factor; Ig=immunoglobulin; anti-CCP3=anti-cyclic citrullinated peptide; IA=inflammatory arthritis; RA=rheumatoid arthritis* | | | |

| Supplemental Table 4. Characteristics of the DoDSR cohort | | | |
| --- | --- | --- | --- |
|  | Women | Men | p-value |
| Number (%) of cohort | 102 (47%) | 113 (53%) | - |
| Age at earliest/baseline sample, mean (SD) | 25 (6) | 25 (6) | 0.863 |
| Age at diagnosis of RA, mean (SD) | 36 (8) | 37 (8) | 0.209 |
| Autoantibody patterns at earliest sample (mean of ~11 years pre-RA diagnosis for women, and ~12 years for men)  anti-CCP3(+), N (%)  anti-CCP3(+) >60, N (%)  RF-IgA(+), N (%)  RF-IgM(+), N (%)  RF-IgA and RF-IgM(+), N (%)  anti-CCP3, RF-IgA and RF-IgM(+), N (%) | 24 (24%)  17 (17%)  **26 (26%)**  18 (18%)  **15 (15%)**  8 (8%) | 24 (21%)  18 (16%)  **15 (13%)**  10 (9%)  **4 (4%)**  3 (3%) | 0.744  **0.025**  0.068  **0.007**  0.121 |
| Autoantibody patterns at immediate pre-RA diagnosis sample (mean of ~0.7 years pre-RA diagnosis for women, and ~1.0 years for men)  anti-CCP3(+), N (%)  anti-CCP3(+) >60, N (%)  RF-IgA(+), N (%)  RF-IgM(+), N (%)  RF-IgA and RF-IgM(+), N (%)  anti-CCP3, RF-IgA and RF-IgM(+), N (%) | 80 (79%)  76 (75%)  58 (57%)  **68 (67%)**  54 (53%)  53 (52%) | 77 (69%)  75 (67%)  61 (55%)  **53 (47%)**  45 (40%)  44 (39%) | 0.089  0.681  **0.004**  0.076  0.098 |
| Autoantibody patterns at post-RA diagnosis sample (mean of ~1.8 years post-RA diagnosis for women, and ~1.2 years for men)  anti-CCP3(+), N (%)  anti-CCP3(+) >60, N (%)  RF-IgA(+), N (%)  RF-IgM(+), N (%)  RF-IgA and RF-IgM(+), N (%)  anti-CCP3, RF-IgA and RF-IgM(+), N (%) | 82 (80%)  79 (78%)  63 (62%)  **70 (69%)**  **56 (55%)**  54 (53%) | 79 (71%)  77 (69%)  61 (55%)  **55 (49%)**  **45 (40%)**  44 (39%) | 0.113  0.332  **0.005**  **0.040**  0.055 |
| Abbreviations: DoDSR=Department of Defense Serum Repository; SD=standard deviation; RF=rheumatoid factor; Ig=immunoglobulin; anti-CCP3=anti-cyclic citrullinated peptide; RA=rheumatoid arthritis | | | |

| Supplemental Table 5. Comparison of women and men within the Healthfair cohort | | | |
| --- | --- | --- | --- |
|  | Women (n=59) | Men  (n=31) | p-value |
| Incident IA/RA, n (%) | 20 (34%) | 6 (19%) | 0.221 |
| Age at baseline visit, mean (SD) | **56 (12)** | **61 (10)** | **0.027** |
| Non-Hispanic white | 48 (81%) | 26 (84%) | 0.524 |
| At least 1 allele with the shared epitope, n (%) | 30 (52%) | 10 (32%) | 0.117 |
| Ever smoker, n (%) | 23 (39%) | 12 (39%) | 1.000 |
| Positive for anti-CCP3 at standard cut-off, n (%) | 59 (100%) | 31 (100%) | 1.000 |
| Positive for anti-CCP3 at >40 units, n (%) | 41 (70%) | 20 (65%) | 0.643 |
| Positive for anti-CCP3 >60 units, n (%) | 28 (48%) | 13 (42%) | 0.661 |
| RF patterns at baseline, n (%)  RF-IgA(-) RF-IgM(-)  RF-IgA(-) RF-IgM(+)  RF-IgA(+) RF-IgM(-)  RF-IgA(+) RF-IgM(+) | 41 (70%)  9 (15%)  2 (3%)  7 (12%) | 25 (81%)  5 (16%)  0 (0%)  1 (3%) | 0.444 |
| Abbreviations: RF=rheumatoid factor; Ig=immunoglobulin; anti-CCP3=anti-cyclic citrullinated peptide; RA=rheumatoid arthritis | | | |

**
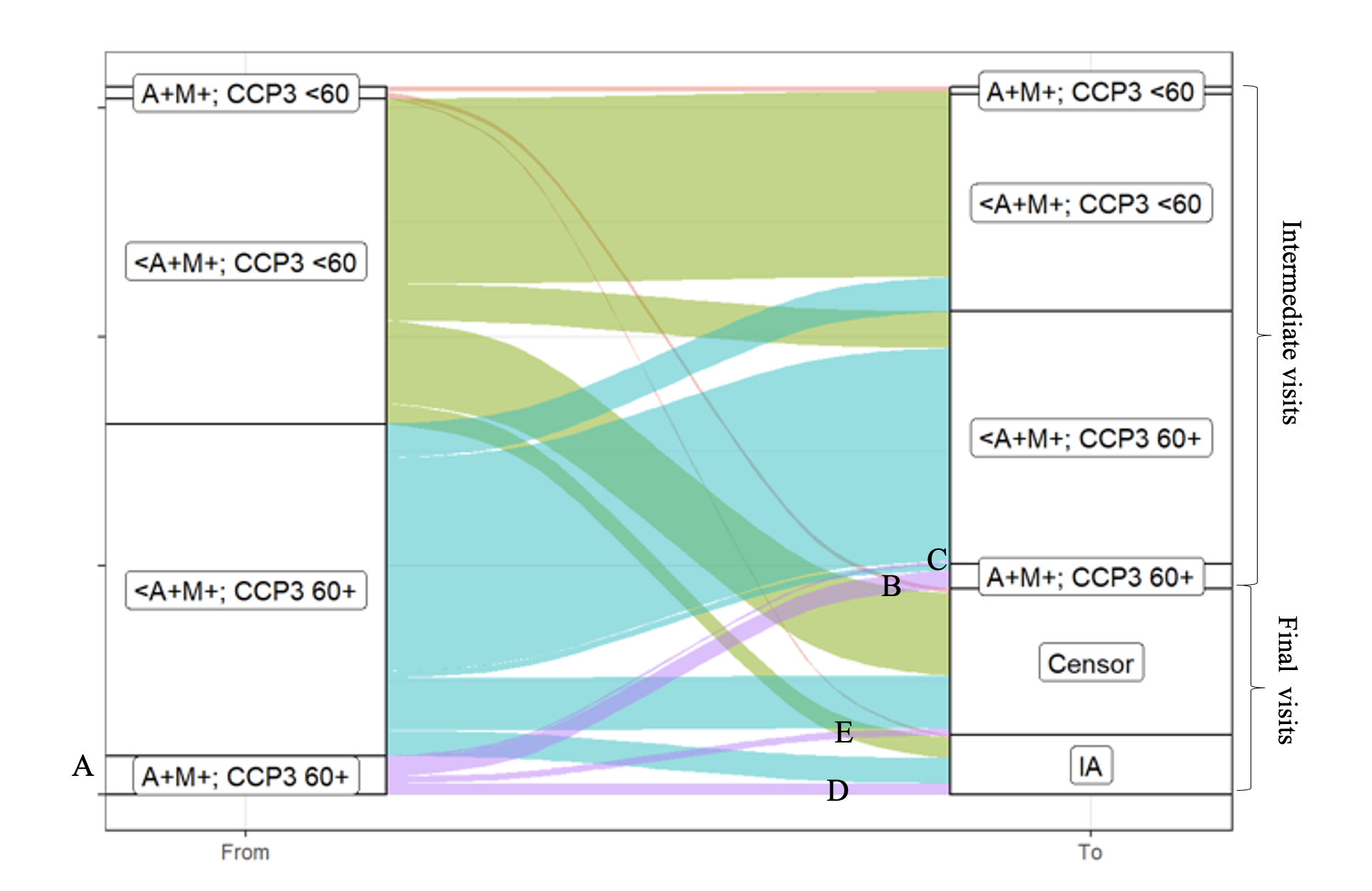
**

**Supplemental Figure 1. Descriptive visualizations of the transitions of autoantibody positivity patterns over time in the Healthfair cohort.** On the left side (’From’) are the autoantibody patterns in all samples from initial visits from all participants (n=90). The colored areas represent the movement of individual samples to the far right column which designates another autoantibody ‘state’ over time (‘To’). The new autoantibody state could be at an ‘Intermediate’ visit meaning that there is another visit after that, and to a ’Final’ visit for those who did not develop incident IA/RA (designated ‘Censor’ on the bottom right, n=64 participants), or the visit immediately prior to incident IA/RA (designated ‘IA’ on the bottom right). As examples, at the bottom left (A), initial samples would be positive for an anti-CCP3 level >60 as well as dual positive for RF-IgA and RF-IgM (A+M+); the purple lines emanating from the left to the right indicate that a portion of those samples continued to be positive for the sample profile at an intermediate visit (B), a small subset lost positivity for one or more RF (C); in addition a portion of ‘triple positive’ samples are ultimately found in individuals who developed incident IA (labelled ‘D’), and a portion was also present in individuals who had not developed IA at their final visit (E). *Abbreviations: A=Rheumatoid factor Immunoglobulin [Ig] A; M=RF-IgM; CCP3=anti-cyclic citrullinated peptide antibody; IA=inflammatory arthritis*
